# A Pore Idea: the ion conduction pathway of TMEM16/ANO proteins is composed partly of lipid

**DOI:** 10.1007/s00424-015-1777-2

**Published:** 2016-01-06

**Authors:** Jarred M. Whitlock, H. Criss Hartzell

**Affiliations:** Department of Cell Biology, Emory University School of Medicine, Atlanta, GA 30322 USA

**Keywords:** Anoctamin, TMEM16, Chloride channel, Phospholipid scrambling, Protein-lipid interactions, Calcium

## Abstract

Since their first descriptions, ion channels have been conceived as proteinaceous conduits that facilitate the passage of ionic cargo between segregated environments. This concept is reinforced by crystallographic structures of cation channels depicting ion conductance pathways completely lined by protein. Although lipids are sometimes present in fenestrations near the pore or may be involved in channel gating, there is little or no evidence that lipids inhabit the ion conduction pathway. Indeed, the presence of lipid acyl chains in the conductance pathway would curse the design of the channel’s aqueous pore. Here, we make a speculative proposal that anion channels in the TMEM16/ANO superfamily have ion conductance pathways composed partly of lipids. Our reasoning is based on the idea that TMEM16 ion channels evolved from a kind of lipid transporter that scrambles lipids between leaflets of the membrane bilayer and the modeled structural similarity between TMEM16 lipid scramblases and TMEM16 anion channels. This novel view of the TMEM16 pore offers explanation for the biophysical and pharmacological oddness of TMEM16A. We build upon the recent X-ray structure of nhTMEM16 and develop models of both TMEM16 ion channels and lipid scramblases to bolster our proposal. It is our hope that this model of the TMEM16 pore will foster innovative investigation into TMEM16 function.

## Introduction

Lipid membranes are energy barriers that charged ions must cross to enter or exit cellular compartments. The major component of this barrier is the energy associated with moving the ion from an aqueous medium of high dielectric constant (*ε* = 80) into a greasy, hydrophobic one composed of lipid acyl chains having a low dielectric constant (ε ~ 4). Ion channels lower this energy barrier by forming a high dielectric (“aqueous”) conduit through the acyl chains. This principle is illustrated beautifully by the structure of KcsA, the first ion channel to have its atomic structure determined by X-ray crystallography in 1998 by the McKinnon lab [67]. At the mouth of the pore, the K^+^ ion loses its bound waters and, as it passes into the selectivity filter, is stabilized by electronegative carbonyl oxygen atoms of the protein backbone. These carbonyl oxygens act essentially as surrogate water molecules that surround the K^+^ ion to provide a hydrophilic environment for the K^+^ ion to traverse the membrane. Since then, KcsA has served as a conceptual icon for ion permeation through proteinaceous pores. Although variations on this theme abound, the idea that ion channels rely entirely on protein-lined pores to facilitate ion passage through the hydrophobic core of the membrane is strongly rooted in our collective concepts of ion channels.

In contrast to our clear understanding of cation channels like KcsA, anion channels remain somewhat enigmatic. For example, prior to the availability of Cl^−^ channel crystal structures, approaches to identify the selectivity filters of the ClC, bestrophin, CFTR, and TMEM16 chloride channels by mutagenesis yielded results that were more ambiguous than those we came to expect from K^+^ channels. Although more clarity has been achieved as some Cl^−^ channel crystal structures have become available, many questions about how Cl^−^ moves through the channel still remain.

### The TMEM16 family

**Fig. 1:**
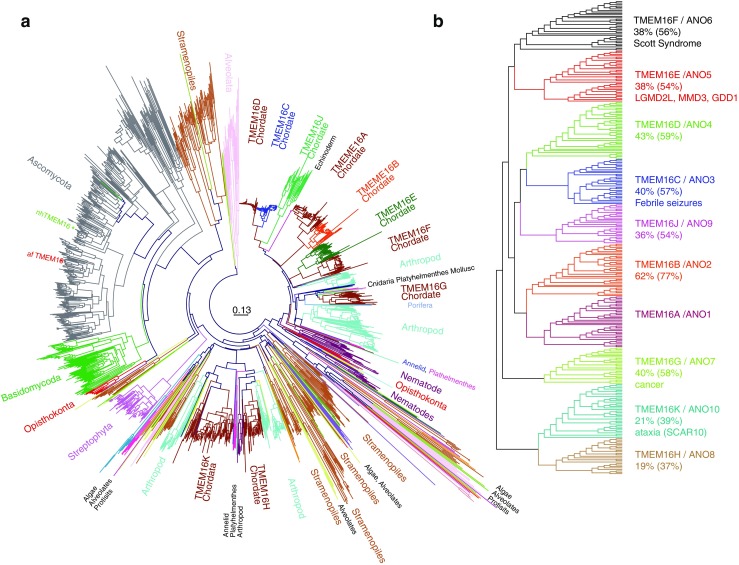
The TMEM16/Anoctamin (ANO) family tree. **a** A phylogenetic tree generated from 1650 TMEM16 sequences in Uniprot. Non-redundant sequences were aligned by MUSCLE [63] and columns containing >50 % gaps were removed with TrimAl [20]. Phylogenetic trees were constructed by CLCBIO Main Workbench 6.9 using Kimura Neighbor-Joining. The fungal TMEM16 proteins afTMEM16 and nhTMEM16 are indicated. **b** A subset of vertebrate TMEM16 proteins identified by Uniprot were assembled and curated to remove splice variants and duplicate sequences. The sequences were truncated by deleting (~50) variable N-terminal amino acids. Trees were displayed using Dendroscope (http://dendroscope.org/). Percent identity and (similarity) refer to human proteins compared to human TMEM16A. Brief description of known disease relevance follows sequence alignments

TMEM16 proteins are found in all eukaryotes (Fig. 1a). In vertebrates, the family consists of ten genes (TMEM16A–TMEM16K with 16I skipped) (Fig. 1b). Although the HUGO-approved name of the family is ANO (“Anoctamin” derived from ANion + OCTA=8 transmembrane domains), most investigators prefer the TMEM16 terminology because many TMEM16 proteins are not anion channels, and they have ten, not eight, transmembrane domains. After TMEM16A and TMEM16B were discovered to be *bona fide* Ca^2+^-activated Cl^−^ channels (CaCCs) in 2008 [21, 98, 127], it was fully expected that all—or most—of the TMEM16 genes would encode CaCCs because of their high sequence similarity (Fig. 1b). The first clue that things might not be so simple came when we were unable to find Cl^−^ currents when we expressed TMEM16C–TMEM16K in HEK cells [27]. This surprising result was soon resolved by the revelation that some TMEM16s are not CaCCs, but are phospholipid scramblases that transport lipids between the two leaflets of the membrane bilayer [17, 108]. This unexpected and exciting discovery that the TMEM16 family is functionally split raises very interesting questions about the evolutionary relationships of TMEM16 channels and scramblases. Below, we will briefly discuss some of the properties of the TMEM16 proteins. This summary is highly selective, and the reader is encouraged to consult several excellent, more balanced reviews [40, 57, 84, 87]. The goal of this article is to develop the *hypothesis* that: (1) TMEM16A evolved from an ancestral lipid scramblase, (2) the TMEM16A pore shares structural similarity to ancestral TMEM16 lipid channels, and (3) the Cl^−^-selective pore of TMEM16A is formed—not of pure protein—but is partly composed of lipids. We will then show how this hypothesis explains a number of enigmatic features of TMEM16A currents. Our hypothesis about the TMEM16A pore draws on a new interpretation of TMEM16F structure function. Although TMEM16F has been shown to be a phospholipid scramblase, it also conducts ions. We will review the TMEM16F literature to develop the idea that the TMEM16F ion conduction pathway is physically the same as the lipid conduction pathway. We then suggest that the homologous pathway in TMEM16A has evolved to conduct ions but not lipids.

### Phospholipid Scrambling

**Fig. 2:**
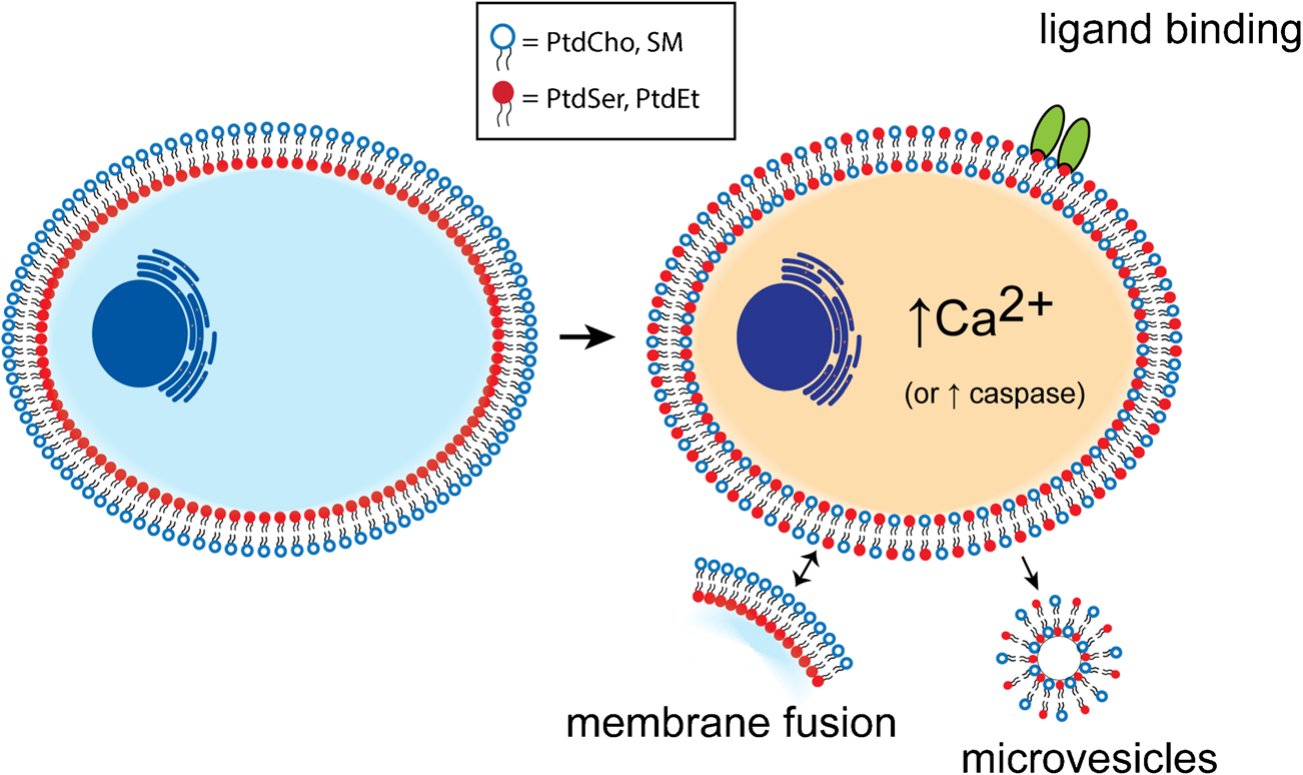
Phospholipid scrambling is a ubiquitous cell signaling process. *Left:* Phospholipids are asymmetrically distributed between the two leaflets of the plasma membrane. PtdCho and sphingomyelin (open blue circles) are concentrated in the outer leaflet while PtdEtn and PtdSer (*solid red circles*) are concentrated in the inner leaflet. *Right:* Phospholipid scrambling stimulated by elevation of cytosolic Ca^2+^ or by apoptotic caspase activation results lipid mixing that exposes PtdSer and PtdEtn on the external leaflet. PtdSer and PtdEt exposure results in assembly of various macromolecular complexes (ligand binding) and membrane trafficking events associated with cell fusion and production of microvesicles [29, 42, 45, 50, 51, 118]

The lipid composition of cell membranes varies, but characteristically, the outer leaflet of the eukaryotic plasma membrane is enriched in phosphatidylcholine (PtdCho) and sphingomyelin (SM), while phosphatidylethanolamine (PtdEt), phosphatidylserine (PtdSer), and phosphatidylinositides are almost exclusively retained in the inner leaflet [70, 119, 122, 123, 132] (Fig. 2). The asymmetric organization of lipid species between leaflets contributes to the physical properties of the membrane, regulates protein function, controls membrane permeability and membrane trafficking, and determines membrane curvature [46, 56, 60, 88, 120, 128]. Cells can disrupt this asymmetry by redistributing lipid species between membrane leaflets by phospholipid scrambling (PLS) [88]. At least two independent pathways activate PLS: One is dependent on caspase activity and the other on Ca^2+^ signaling [10, 11, 29, 55, 107]. One important consequence of lipid scrambling is the exposure of negatively charged lipid species like PtdSer on the external leaflet of the membrane [11, 55]. Exposed PtdSer acts as a platform for assembly of signaling complexes and plays an essential role in a variety of cellular functions including apoptotic cell recognition by phagocytes [29], the activation of blood platelets where it serves as a catalytic scaffold for the assembly of coagulation factors [9, 10, 62], and the fusion of progenitor cell types to produce multinucleated cells (e.g. muscle fibers [45, 51, 118]) (Fig. 2).

### TMEM16 proteins have diverse functions and many are linked to PLS

Although it was initially assumed that all TMEM16s were CaCCs because of their high sequence similarity and predicted transmembrane topologies, so far only TMEM16A and TMEM16B have been unambiguously shown to be CaCCs [84, 113]. Although it is possible that other TMEM16s may function as CaCCs, possibly in intracellular locations [27], there is growing appreciation for the diverse molecular functions of the TMEM16 family. TMEM16C (57 % similarity to TMEM16A) has been identified as a β-subunit of the Na^+^-activated potassium channel SLACK and has been linked to febrile seizures but elicits no ion channel activity on its own [30, 48]. TMEM16E (54 % similarity to TMEM16A) has been linked to several types of muscular dystrophy [13, 43, 85, 97] and bone disease [115], but the molecular function of TMEM16E has not yet been identified. TMEM16F (56 % similarity to TMEM16A) elicits Ca^2+^-activated phospholipid scrambling (Ca^2+^-PLS) and mutations in the protein cause the congenital bleeding disorder Scott’s Syndrome [108, 130, 131]. TMEM16G (58 % similarity to TMEM16A) has been suggested to be a cell-cell junction protein in prostate tissue and has been recognized for its potential as a prostate cancer biomarker and immunotherapy target, but its function remains in question [23, 24, 77]. TMEM16K (39 % similarity to TMEM16A) mutations cause a type of spinocerebellar ataxia (SCAR10) associated with coenzyme Q10 deficiency, but the exact function of this protein remains to be elucidated [7].

There is now growing suspicion that Ca^2+^-PLS might be a common theme underlying the diverse functions of the TMEM16 proteins. TMEM16C, D, F, G, or J have been reported to elicit Ca^2+^-PLS [106], suggesting that many of the mammalian TMEM16s may be phospholipid scramblases. Recent studies on TMEM16 homologs in evolutionarily distant species that have only one or two TMEM16 genes have bolstered the idea that a unifying feature of the TMEM16s may be their relationship to lipids. Specifically, the purified and reconstituted solitary TMEM16s from the saprophytic fungi *Nectria haematococca* and *Aspergillus fumigatus* both function as phospholipid scramblases [17, 68]. The yeast *Saccharomyces cerevisiae* has one TMEM16, IST2, that is essential for the formation of cortical ER, a structure that plays vital roles in the trafficking of lipids from the endoplasmic reticulum to the plasma membrane [69, 105, 124]. The nematode *Caenorhabditis elegans* has two TMEM16s, one of which is implicated in PLS [64]. The observation that a common function of TMEM16s, especially in “lower” eukaryotes, is lipid-related suggests the possibility that lipid transport may have evolutionarily preceded ion channel function in TMEM16s. In any case, the finding that TMEM16s expressed in such evolutionarily diverse species as human, worm, and fungi function as lipid scramblases suggests the possibility that the entire TMEM16 family—regardless whether they are scramblases or Cl^−^ channels—may have a specific relationship with lipids.

### Phospholipid scrambling by TMEM16F and homologs

TMEM16F was identified for its essential role in Ca^2+^-PLS in an expression-cloning strategy aimed at identifying proteins essential for PLS [108]. Mutations in TMEM16F have been identified as the cause of Scott’s Syndrome [22, 108], a congenital bleeding disorder caused by the loss of Ca^2+^-PLS [131]. Knockout of TMEM16F expression in mice recapitulates the suppression of platelet activation and increased bleeding time observed in Scott’s Syndrome patients [126], and primary cells isolated from TMEM16F^−/−^ mice lack Ca^2+^-PLS [106]. Exogenous expression of TMEM16 C, D, F, G, or J rescues Ca^2+^-PLS in cells isolated from TMEM16F^−/−^ mice, but expression of TMEM16 A, B, E, H, or K does not [106]. Further support for the hypothesis that TMEM16F is a scramblase was provided by our identification of a scramblase domain (SCRD) in TMEM16F that when mutated abolishes Ca^2+^-PLS [130]. Replacing the homologous sequence of TMEM16A with the corresponding SCRD of TMEM16F causes TMEM16A to elicit Ca^2+^-PLS.

There is not, however, universal agreement that TMEM16F is a phospholipid scramblase. Some investigators maintain that TMEM16F is an ion channel [36, 72, 100, 126]. Further, Yang et al. [126] were unable to elicit PLS in HEK cells transfected with TMEM16F, in contrast to our results [130]. We also have been somewhat reluctant to conclude definitively that TMEM16F is the scramblase enzyme because we found a weak correlation between TMEM16F expression level and PLS activity [130]. However, with the demonstration that purified fungal TMEM16s function as phospholipid scramblases in reconstituted systems [17, 68], it seems hard to avoid the conclusion that certain TMEM16s are phospholipid scramblases.

The mechanism by which lipids traverse the plasma membrane remains an outstanding question. Ca^2+^-PLS does not require ATP, unlike the flippase and floppase P4-ATPases and ABC transporters that do [71, 96]. Rather, phospholipid scrambling is thought to be driven by downhill flux of lipid from high concentration in one leaflet to low concentration in the other. Scramblases must work differently than ion channel pores because the permeant molecule (lipid) is amphipathic rather than purely hydrophilic: The routes taken by the hydrophobic acyl chains and the hydrophilic head groups must be environmentally different. Thus, the conduction pathway must somehow accommodate the amphipathic nature of its cargo. One solution would be a hydrophilic furrow in the protein that would allow the head groups to translocate from one side of the membrane to another while the acyl chains remain in the hydrophobic phase of the membrane. Such a model was proposed in 2006 by Pomorski and Menon [88].

**Fig. 3:**
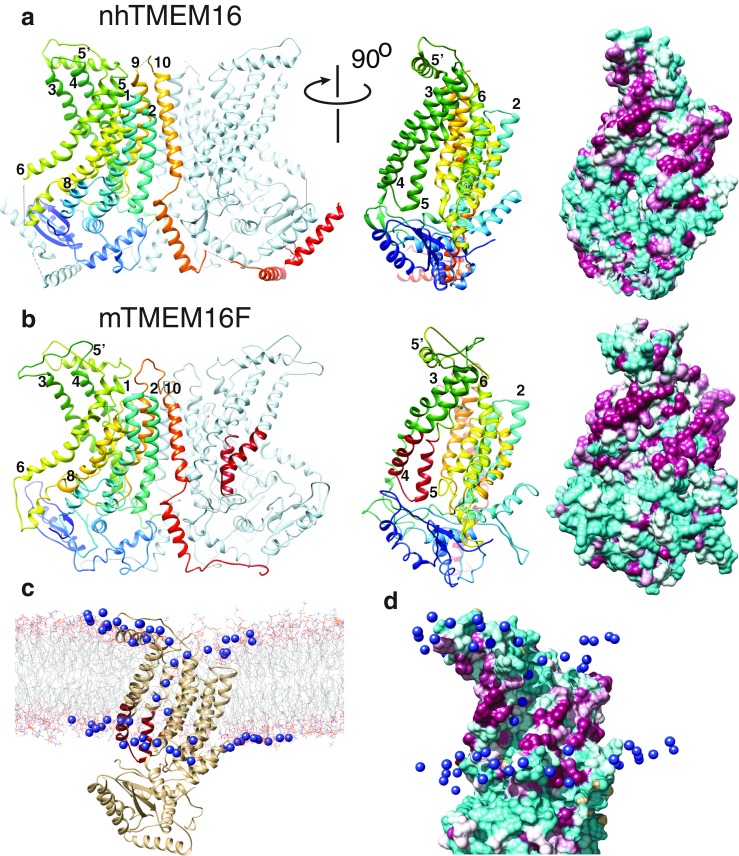
Phospholipid scrambling by TMEM16 proteins. **a** Crystal structure of nhTMEM16 [17] (4WIS) and **b** a homology model of TMEM16F based on the nhTMEM16 structure using Phyre2 [54]. One monomer is colored rainbow (*blue* is N-terminus, *red* is C-terminus) and the other is *grey- light blue*. Helices are numbered. *Left panels*: dimer viewed from the plane of the membrane. *Middle panels*: one monomer rotated 90° around the *y*-axis. The scrambling (SCRD) domain in TMEM16F in B is colored *firebrick red. Right panels:* molecular surfaces of the same view as the middle panel. *Cyan* = hydrophilic (−4.5, Kyte-Doolittle scale). *Magenta* = hydrophobic (4.5). **c** Molecular dynamics simulation of interaction of lipids with nhTMEM16 (http://sbcb.bioch.ox.ac.uk/memprotmd/beta/protein/pdbid/4WIS). A bilayer-embedded model was produced from the nhTMEM16 crystal structure through the MemProtMD protocol [104]. Lipids are shown in wire representation and the nitrogens of the choline headgroups of PtdCho molecules near the protein are shown as *blue spheres*. PtdCho molecules can be seen in the hydrophilic furrow and clustering near the SCRD. **d** Molecular surface of a close-up view of the hydrophilic furrow. The orientation is the same as **c**. Only the N of the PtdCho choline head groups is shown. Images were created using UCSF Chimera v. 1.10

Recently, crystallization of a fungal TMEM16 lipid scramblase has provided validation of this model [17] (Fig. 3). The protein has a hydrophilic furrow facing the lipid bilayer that is bordered by helices 4 and 6 and connects the cytosolic and extracellular nhTMEM16 domains (Fig. 3a, middle panel). Intuitively, this furrow is well-suited for transport of lipids from one leaflet to the other. Mark Samson’s laboratory has performed molecular dynamics simulations of nhTMEM16 in a PtdCho bilayer (http://sbcb.bioch.ox.ac.uk/memprotmd/beta/protein/pdbid/4WIS) and has shown that lipid head groups are predicted to populate this furrow, as one would expect if this were a phospholipid conduit across the membrane (Fig. 3c, d). Homology models of TMEM16F show a similar hydrophilic furrow (Fig. 3b). One side of this furrow is lined by the SCRD we recently identified [130].

### TMEM16A may have evolved from phospholipid scramblases

We hypothesize that the TMEM16A Cl^−^ channel evolved from an ancestral phospholipid scramblase. The TMEM16 family is functionally split, with TMEM16A and TMEM16B being Cl^−^ channels and TMEM16C, D, F, and J being scramblases (Fig. 1). This functional duplicity is reminiscent of other anion channels that apparently evolved from transporters. CFTR (cystic fibrosis transmembrane conductance regulator) is a Cl^−^ channel that evolved from ABC transporters [33, 52, 76], and the CLC chloride channels CLC-1, CLC-2, CLC-Ka, and CLC-Kb are members of a 9-gene family that includes five H^+^-Cl^−^ exchangers [65, 75]. It seems reasonable to speculate that the primordial TMEM16 was a phospholipid scramblase and that “broken” versions of the lipid pathway evolved to become Cl^−^ channels. Another piece of evidence supporting the idea that PLS is an ancient function of TMEM16s is the finding that the solitary TMEM16 gene in two different fungi encodes phospholipid scramblases [17, 68].

Regardless whether TMEM16 Cl^−^ channels evolved from lipid scramblases or scramblases evolved from Cl^−^ channels, the sequence similarity of these proteins suggests that both channels and scramblases interact with lipids in particular ways. Within their transmembrane domains (which would be the likely sites of protein-lipid interaction) the Cl^−^ channel TMEM16A is 51 % *identical* to the phospholipid scramblase TMEM16F. Moreover, the finding that we can convert TMEM16A into a scramblase by replacing ~15 amino acids of TMEM16A with aligned amino acids of TMEM16F [130] emphasizes that the architectures of the two proteins are likely very similar.

### The proteolipidic pore hypothesis

**Fig. 4:**
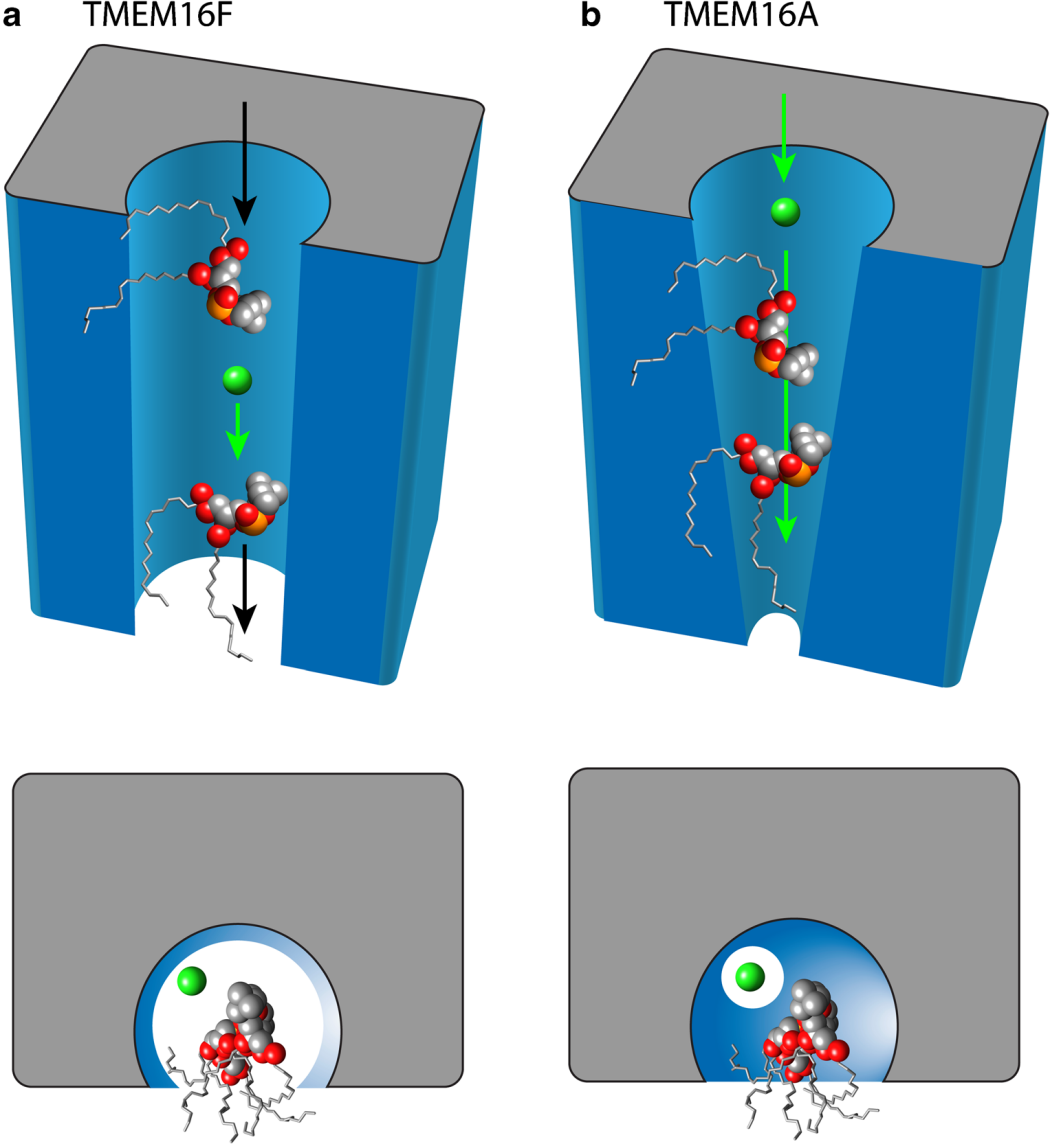
Hypothesis for evolution of a Cl^−^ channel from a phospholipid scramblase. As discussed in the text, we believe that PLS mediated by TMEM16F is associated with leakage of ions through the lipid scrambling pathway (the hydrophilic furrow) between the protein and the scrambling lipid head groups. Structural changes in the phospholipid scrambling pathway during evolution may have produced a Cl^−^ selective channel by decreasing phospholipid mobility in the furrow while still retaining the association of the head groups with the protein as a structural component. In this case, ions would still be capable of flowing between the lipid head groups and the protein. Ionic selectivity would be determined by both the protein and cognate phospholipids. **a** Cartoon of the scrambling furrow showing TMEM16F scramblase. *Top panel* is viewed in perspective from the plane of the membrane. *Bottom panel* is a view down the furrow from the extracellular space. Two PtdCho molecules are shown moving through furrow along with a Cl^−^ ion. The phospholipids are shown with their acyl tails in stick representation projecting into the hydrophobic bilayer. The polar head group atoms are shown as spheres with the atoms colored by element (*grey* = carbon, *red* = oxygen, *orange* = phosphorous). **b** Cartoon of TMEM16A. Two PtdCho molecules are lodged in the furrow because it is too narrow for them to move. However, Cl^−^ ions can slip between the lipid head groups and the protein. The effective diameter of the “pore” is imagined as smaller in TMEM16A than TMEM16F as seen in the lower panels. Although only 2 phospholipid molecules are shown, we calculate that the furrow is filled with 4–5 phospholipid molecules creating a monolayer that joins the outer and inner leaflets

If the architectures of TMEM16A and TMEM16F are alike, their interactions with lipid are probably similar, even though TMEM16A does not appear to transport lipids. From mutagenesis experiments on mammalian TMEM16F [130] and the X-ray structure of the *N. haematococca* TMEM16 phospholipid scramblase [17], there is good reason to believe that lipid scrambling occurs by a flipping of lipid head groups through the hydrophilic furrow running between the cytosolic and extracellular domains of the TMEM16 protein. As described in more detail below, ionic currents that have been associated with TMEM16F are likely leak currents flowing through this furrow concurrent with phospholipid movement. We suggest that, in TMEM16A, the Cl^−^ permeation pathway is structurally cognate to the hydrophilic furrow in TMEM16F. This idea is illustrated in Fig. 4 which shows how we imagine ions and lipids move in TMEM16F and TMEM16A. We suggest that TMEM16 Cl^−^ channels might have evolved from scramblases if lipid scrambling activity were lost while the ionic leak pathway was retained in some form. This could have occurred in several ways. The hydrophilic furrow could have folded over and narrowed, essentially converting the furrow into a pore that only ions but not lipids could pass. However, formation of an enclosed aqueous channel would presumably require major structural rearrangements. Alternatively, less drastic molecular rearrangements might have conspired to create an aqueous pore if lipid head groups were included as part of the ion conduction pathway. We propose that the TMEM16A protein stabilizes a non-bilayer phase in the membrane so that the two leaflets are continuous (⊃) where they interact with the protein. However, the furrow in TMEM16A is partially obstructed, disallowing the lipids to move through the furrow. The lipid head groups would then provide a hydrophilic environment forming half of the pore and ions could move across the membrane in the “channel” formed between the protein and the lipid head groups. Just as the ionic currents flowing during lipid scrambling in TMEM16F likely represent leak of ions around the lipid-protein interface, the ions would flow through TMEM16A in the analogous space with the lipids playing a structural role. This proposed pore structure explains a number of unusual features of the TMEM16A currents, especially their pharmacology and ionic selectivity.

### TMEM16F conductance is a non-selective leak through the hydrophilic furrow

Our reasoning that the TMEM16A ionic current occurs through a proteolipidic pore is partly inspired by the conclusion that ionic currents in TMEM16F represent leak of ions through the space between the scrambling lipid and the protein. The nature of the TMEM16F ionic current has been a matter of considerable confusion in the literature. It has been reported to be a non-selective cation channel [1, 126], a swelling-activated Cl^−^ channel [2], an outwardly rectifying Cl^−^ channel [72], a CaCC [53, 100, 109], and a CaCC of delayed activation [36]. We think that this diversity of opinion is explained partly by the parable of six blind men describing an elephant: Overall, the data support the conclusion that TMEM16F has a non-selective pore. However, additionally, this diversity may be explained if the TMEM16F pore can exist in multiple open conformations, a restricted conformation and a dilated conformation. In the dilated conformation, the pore is likely to accommodate ions as large as NMDG^+^ and aspartate having a minimum diameter of ~6 Å. Such dilation might be more easily accomplished in a channel with a flexible relationship with lipids than in a proteinaceous pore that is constrained by the secondary and tertiary structure of the protein. There is precedent for this idea: It has been suggested that P2X, ASIC, and certain TRP channels have at least two distinct open states, a restricted and a dilated state. For example, in TRPA1 the restricted state is a non-selective cation channel, whereas the dilated state allows flux of much larger molecules (MW>500) [12].

#### TMEM16F conducts anions

Studies that have concluded that TMEM16F is a Cl^−^ channel show clearly that it conducts anions, but cation permeability was not rigorously examined. For example, Martins et al. [72] suppose that TMEM16F is a Cl^−^ channel because the current is blocked by classical anion channel blockers and because a mutation (Y405F) in the putative pore domain shifts the IV curve as if the relative cation/anion permeability increased. However, the authors do not test whether the E_rev_ of the wild-type TMEM16F current changes with [Cl^−^] [2] according to the GHK equation nor do they analyze quantitatively how the Y405F mutation alters cation/anion permeability. Shimizu et al. [100] argue that TMEM16F is Cl^−^-selective because switching from Na^+^ to NMDG^+^ does not alter E_rev_. However, this conclusion depends on the assumption that NMDG^+^ is impermeant, which other authors contest (see below). Grubb et al. [36] assume that TMEM16F is an anion channel, despite their finding that P_Na_/P_Cl_ = 0.3, because they believe that Na^+^ does not utilize the same pore as anions. They reason that Na^+^ takes a different path than anions because TMEM16F exhibits selectivity among anions: selectivity follows the Eisenman type 1 sequence SCN^−^ > I^−^ > Br^−^ > Cl^−^. They assume that such a selectivity filter would likely exclude cations. In Eisenman’s theory [28], selectivity sequences are the result of two competing phenomena, the attraction of the ion into a charged binding site, and the energetic dehydration penalty for entering the binding site. The type 1 series implies that permeability is dominated by the second of these phenomena; the relative energetic ease with which waters are removed from the ion. While such a mechanism may explain anion selectivity, it does not rule out cations permeating by the same pathway. Indeed, because the ionic radius of Na^+^ is about half that of Cl^−^ (1.0 vs 1.8 Å) and it is even possible that Na^+^ could permeate the channel in a partially hydrated state.

#### TMEM16F also exhibits significant cation permeability

Although TMEM16F may exhibit selectivity among anions, four different labs that have examined cation permeability conclude that TMEM16F also conducts cations, although there is considerable disagreement quantitatively. P_Na_/P_Cl_ has been measured to be 0.3 [36], ~0.5 [99], 1.4 [130], and 6.7 [126]. Indeed, Yang et al. [126] labeled TMEM16F a Ca^2+^-activated non-selective cation channel because the reversal potential does not shift when NaCl is replaced with Na-MES, but this conclusion depends on the assumption that MES^−^ is impermeant, which, as discussed below, may be untrue. In any case, the 20-fold range in P_Na_/P_Cl_ ratios reported in the literature suggests that TMEM16F has a highly variable personality. We contend that one explanation for this may be related to the lipid composition of the membrane. Because the ionic current activates coincidently with phospholipid scrambling [130], the biophysical features of the current may depend on which lipids happen to be scrambling and the charge of the lipid head groups that are engaged.

#### TMEM16F has a large pore

Two labs have found that NMDG^+^ is permeant through TMEM16F channels: Yu et al. [130] find P_NMDG_/P_Cl_ = 0.5, while Yang et al. [126] report P_NMDG_/P_Cl_ ~ 2. NMDG^+^ has dimensions of 5.5 Å × 6.0 Å × 11.7 Å and an estimated minimum diameter of 6 Å [12]. Furthermore, two labs report that the channel is significantly permeable to aspartate (P_Asp_/P_Cl_ = 0.5), which has an estimated minimum diameter of 5.1 Å [36, 100]. If TMEM16F is permeable to NMDG^+^, it is likely also permeable to MES^−^, which has the same molecular mass. If MES^−^ is permeant, this might explain why Yang et al. [126] conclude TMEM16F is a cation channel. In contrast, Grubb et al. (2013) propose that NMDG^+^ is impermeant because E_rev_ = E_Cl_ with 100 mM Cs-aspartate + 40 mM CsCl inside and 140 NMDG-Cl outside and the IV curve is described by the GHK equation [36]. However, the conclusion might be compromised if NMDG^+^ and Cs^+^ are also permeable. Our review of the literature leads us to conclude that TMEM16F is relatively ion non-selective and that the pore diameter may be >6 Å. This argument leads us to propose that TMEM16F ionic currents are caused by a leak that occurs while lipids are being transported (Fig. 4a). As reviewed above, lipid transport in nhTMEM16 is thought to occur via the hydrophilic furrow that is bordered by TMD4 and TMD6. We propose that ions also flow through this pathway coincident with lipid movement.

### Fungal afTMEM16 has a large, lipid-dependent, non-selective ion conductance

afTMEM16 reconstituted into lipid bilayers exhibits a single channel conductance of ~300pS and is poorly ion-selective (P_K_:P_Cl_ = 1.5). The estimated pore diameter is 8–13 Å [68]. Malvezzi et al. suggest that afTMEM16 has separate pathways for ions and lipid because they can separate PLS and ionic currents. For example, ionic currents were not observed when afTMEM16 was reconstituted with a lipid mixture of POPE/POPG (3:1), whereas currents were observed in more complex lipid mixtures containing *Escherichia coli* polar lipids [68]. In contrast, PLS was similar regardless of lipid composition. These studies further showed that PLS was not dependent on ionic current by replacing K^+^ with presumably impermeant NMDG^+^. This ability to separate ionic currents from PLS suggests that the ions and lipids take different pathways. However, an equally probable explanation is that, depending on the species of lipid present, the conformational packing of the protein and lipid differs in a manner that essentially seals the lipid transport pathway so that ionic leak is minimized. In contrast to afTMEM16, Brunner et al. report that nhTMEM16 does not exhibit ionic currents when reconstituted in *E. coli* polar lipids and egg PtdCho [17]. One possible explanation of this result is that the lipids chosen for reconstitution may have not been the “correct” ones that support nhTMEM16 ionic currents. Brunner et al. also were unable to find currents when they expressed nhTMEM16 in HEK cells, but the protein did not appear to traffic well to the plasma membrane.

### Cl^−^ conduction through TMEM16A occurs via the hydrophilic furrow

**Fig. 5:**
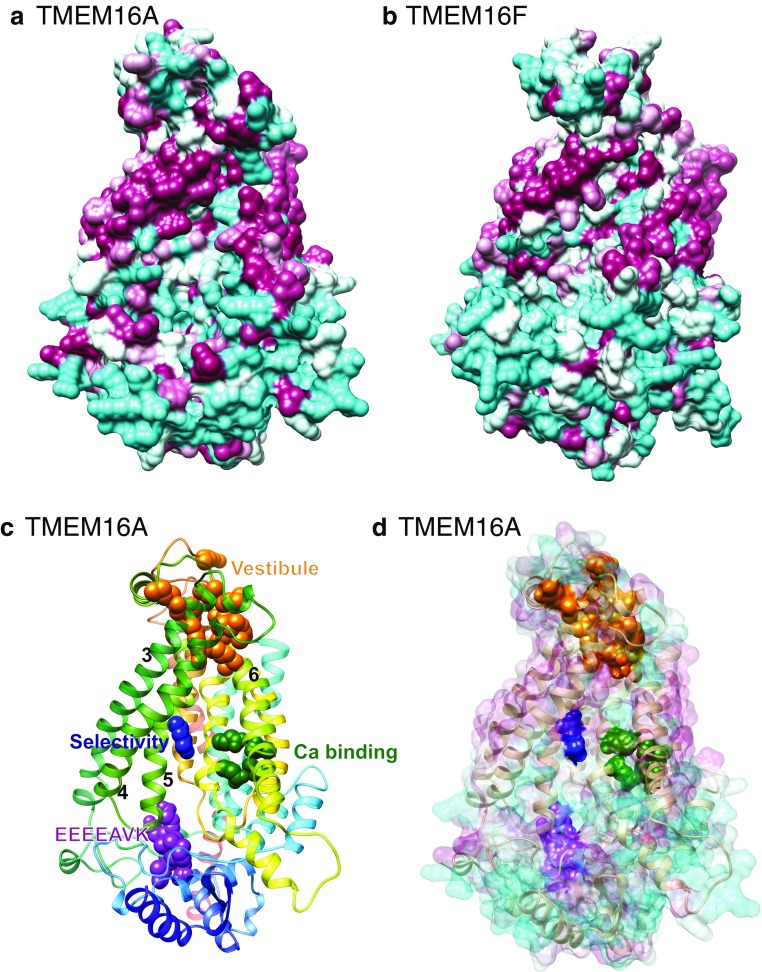
The TMEM16A furrow likely forms the conduction pathway for Cl^−^. Molecular surface of homology models of **a** TMEM16A and **b** TMEM16F. Homology models were made from nhTMEM16 structure (4WIS) using Phyre2 [54]. *Cyan* = hydrophilic (−4.5, Kyte-Doolittle scale). *Magenta* = hydrophobic (4.5). Note hydrophobic region at the base of the furrow in TMEM16A that is hydrophilic in TMEM16F. **c** Functional residues of TMEM16A. Homology model of TMEM16A with functional amino acids identified by mutagenesis shown as spheres. *Orange*: vestibule [86, 129]. *Blue*: selectivity [126]. *Green*: Ca^2+^ binding [126, 129]. *Magenta*: gating modifier EEEEEAVK [125]. Transmembrane helices are colored as in Fig. 3. **d** Model of TMEM16A with a superimposed surface colored by hydrophobicity showing the relationship of the functional residues to the hydrophilic cleft

We constructed a homology model of TMEM16A based on the fungal nhTMEM16 structure. Although the extracellular and intracellular domains are not well modeled, the transmembrane domains are modeled with high confidence [130]. These models show that TMEM16A has a hydrophilic furrow that resembles nhTMEM16 (and our TMEM16F homology model) with the exception of the presence of a hydrophobic patch at the cytoplasmic end of the furrow (Fig. 5a, b). This patch might explain why TMEM16A is not a scramblase because these hydrophobic amino acids might provide a barrier for movement of the hydrophilic head groups through the furrow.

Mutagenesis of TMEM16A has revealed a number of amino acids that participate in the Cl^−^ conduction pathway. Below, we summarize these data and show that it is likely that these residues are located along the hydrophilic furrow. Using cysteine accessibility mutagenesis, we identified a number of amino acids between G628 and Q637 in TMEM16A that we predicted comprise the vestibule of the pore [129]. We considered that these amino acids were located in the vestibule because cysteine-reactive MTS reagents were able to alter current density and anionic selectivity in cysteine-substituted TMEM16A channels. In addition, Peters et al. [86] identified 4 basic residues equivalent to R515, K603, R621, and R788 that alter anion selectivity similarly to those we found in the vestibule. The mutations also change the sensitivity of TMEM16A to two newly described blockers, NTTP and 1PBC, that block the channel in a weakly voltage dependent manner (δ ~ 0.1). When mapped onto an TMEM16A homology model based on the nhTMEM16 structure, all of these amino acids cluster at the top of the hydrophilic furrow that is analogous to the presumptive lipid transport furrow in nhTMEM16 (Fig. 5c, d, orange spheres). These data strongly indicate that the Cl^−^ conduction pathway of TMEM16A includes the hydrophilic furrow.

Another residue that has been implicated in TMEM16A ionic selectivity is K588 [126]. This amino acid is located at the N-terminus of the SCRD-homology domain that we identified and lies in the hydrophilic furrow [130] (Fig. 5c, d, dark blue spheres). The K588Q mutation almost doubles the relative Na^+^ permeability (P_Na_/P_Cl_ increases from 0.14 to 0.24).

The Ca^2+^ binding site that is responsible for activation of TMEM16A is also located at one side of the hydrophilic furrow [114, 130] (Fig. 5c, d, green spheres). This suggests that Ca^2+^ may gain access to its binding site by entering the hydrophilic furrow from the cytosolic side. This may explain why the current is outwardly rectifying at low Ca^2+^ concentrations: negative voltages may effectively pull Ca^2+^ from its binding site. In any case, the location of the Ca^2+^ binding site in the hydrophilic furrow supports the idea that this is a hydrophilic environment that is energetically favorable for occupation by ions.

Finally, we showed that the EEEEEAVK sequence in the first intracellular loop between TMD2 and TMD3 is involved in allosterically regulating channel gating and Ca^2+^ sensitivity [125]. A similar conclusion was reported by Ferrera et al. [31]. This sequence is located at the cytosolic end of the hydrophilic furrow (Fig. 5c, d, magenta spheres).

Taken together, these functional residues define the hydrophilic furrow and suggest that Cl^−^ ions traverse the membrane by way of this furrow. However, this suggestion raises a conundrum because the furrow is essentially a hemi-channel with its open side facing the core of the membrane. If the core of the membrane is composed of hydrophobic lipid acyl chains, as would be the case if the lipid is structured as a bilayer, it is not clear how the furrow would provide a sufficiently hydrophilic environment for Cl^−^ ions to move. To solve this problem, we propose that the open side of the furrow is filled with hydrophilic lipid head groups that have their acyl chains oriented approximately parallel to the membrane (rather than perpendicular to the membrane as in a phospholipid bilayer). In TMEM16F, we imagine that movement of lipids from one leaflet to the other involves a lipid head groups slipping along the hydrophilic furrow in tight association with one another. In effect, we think that the lipids form a continuous monolayer connecting the inner and outer leaflets with no gaps. The head groups are oriented towards the protein in the furrow while the acyl chains dangle in the hydrophobic phase. In TMEM16A, we imagine that the lipid head groups occupy equivalent locations, but they do not exhibit net movement (scrambling does not occur) due to the inability of the furrow to dilate sufficiently to allow the passage of lipid head groups from one leaflet to another. In our conceptualization, for this furrow to function as a proteolipidic Cl^−^ selective pore, the lipid head groups must be well ordered, tightly juxtaposed to one another, and in close association. In other words, the polar head groups must completely fill the open face of the furrow, as any gaps would expose permeant ions to the hydrophobic acyl chains that occupy the inner membrane space. Indeed, the amphipathic nature of phospholipids is perfectly suited to form a hydrophilic channel in the lipid bilayer with little energetic penalty for the ion passing along the polar head groups. Despite the restricted dilation of TMEM16A that disallows the exchange of lipid head groups between leaflets, we fancy that there is enough space for Cl^−^ ions to slip between the protein and the lipid head groups to traverse the membrane (Fig. 4b).

**Fig. 6:**
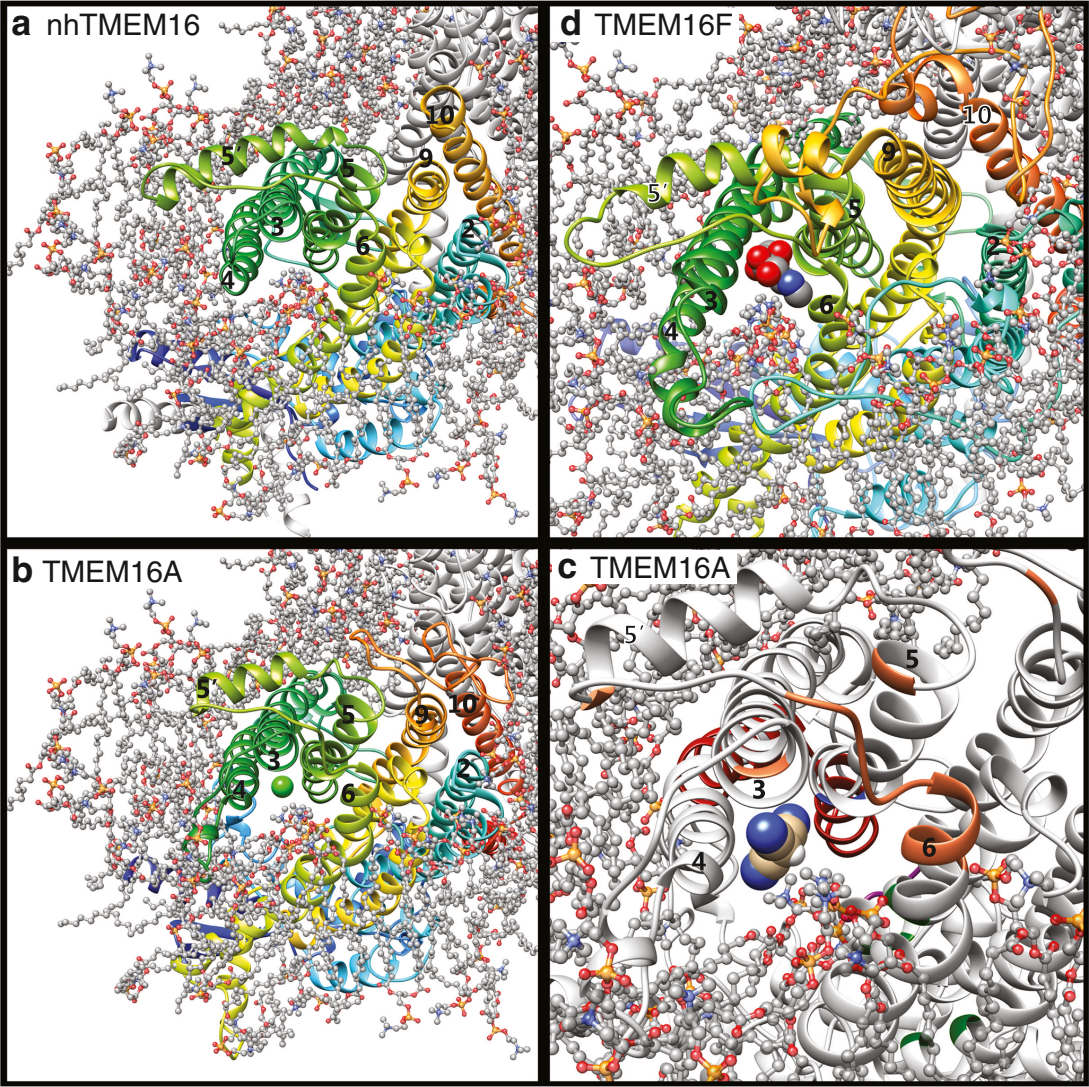
Lipid head groups may form part of the Cl^−^ conductance pathway in TMEM16A. **a** Molecular dynamics simulation of nhTMEM16 in a PtdCho bilayer, viewed from the extracellular space looking down the hydrophilic furrow formed by transmembrane helices α3, α4, α5, and α6. PtdCho molecules are shown in ball-and-stick representation colored by element (C=*grey*, *blue*=N, P=*orange*, O=*red*). Lipid head groups are seen in the furrow. Helices are numbered and colored as in Fig. 3. **b–d** Fantasy models of how ions may permeate TMEM16A and TMEM16F. **b** An homology model of TMEM16A was placed in register with nhTMEM16 using MatchMaker in UCSF Chimera with the Needleman-Wunsch alignment algorithm. The lipids were kept in the same absolute position as in A. The vestibule residues are *red* [129] and *orange* [86]. The Cl^−^ ion (*green*) was added to scale to show that it can fit between the lipid head groups and the protein. **c** Tricyanomethanide (C(CN_3_)) was positioned manually in the TMEM16A homology model. Functional residues are colored as in Fig. 4: the SCRD-homology domain [130] formed by α4 and α5 is *red* and the vestibule residues are *orange*. Although largely obscured by other regions of the protein, the selectivity filter is *blue*, the EEEEAVK sequence is *magenta*, and the Ca^2+^ binding residues are *green*. **d** NMDG^+^ was placed manually in the pore of the TMEM16F homology model

We have played with this whim by asking whether it might be possible to construct a TMEM16A pore that is partly composed of lipid. We began by superimposing the homology model of TMEM16A on the molecular dynamics simulation of nhTMEM in a phospholipid bilayer (introduced in Fig. 3c, d). In this pseudo-simulation, the lipids that populate the hydrophilic furrow in nhTMEM16 are found along the analogous structure in TMEM16A (Fig. 6a, b). Interestingly, there is just sufficient space for a Cl^−^ ion to fit between TMEM16A and the lipid head groups located in the furrow. Furthermore, even C(CN)_3_, which we have shown can permeate TMEM16A CaCC channels [92], fits in this space (Fig. 6c). When we superimpose the TMEM16F homology model on the MD simulation, we see that the space between the lipids and the furrow in TMEM16F is even larger than it is in TMEM16A. In TMEM16F, this space allows for NMDG^+^ to fit (Fig. 6d), which is consistent with the lesser selectivity of TMEM16F compared to TMEM16A. These manipulations by no means provide a test of our hypothesis, but simply illustrate the possibility that hydrophilic head groups of lipids could form one side of the TMEM16A pore.

In nhTMEM16 and the TMEM16A and TMEM16F homology models, there are no obvious ion conduction pathways other than this one along the furrow. Although there is a large cavity at the dimer interface formed by TMD 3, 5, and 9 on monomer A and TMD 10 of monomer B viewed from the extracellular side of the membrane, this cavity is highly hydrophobic, suggesting that it is unlikely to be the ion conducting pore.

One potential glitch to this interpretation is our observation that a chimeric protein composed of TMEM16A with the 35-amino acids scrambling domain of TMEM16F can exhibit both Cl^−^ selective currents like TMEM16A and non-selective currents like TMEM16F [130]. At low [Ca^2+^], this chimera exhibits Cl^−^ selective currents similar to TMEM16A, but as [Ca^2+^] is increased further and Ca-PLS is activated, the currents become non-selective like TMEM16F currents. This finding suggests that the chimeric TMEM16A/F pore can exist in different conformations that allow passage of different sized cargo (Cl^−^ ion vs lipid head group). We surmise that the conformation associated with lipid exchange between leaflets involves dilation of the furrow to allow the passage of a wide range of ionic cargo. In contrast, TMEM16A is unable to dilate sufficiently to allow lipid head group passage but is dilated sufficiently to allow the passage of Cl^−^ and possibly some larger ions.

### TMEM16A has low ionic selectivity

One might expect that a proteolipidic pore constructed in such a way would have relatively low selectivity. Although TMEM16A is colloquially called a Cl^−^ channel, it seems that it is often not especially selective. We have previously shown that CaCC channels encoded by TMEM16A are permeable to virtually all monovalent anions <6 Å in diameter, which includes organic anions as large as tricyanomethanide, C(CN)_3_. The relative anionic permeability is related to the energetic penalty of dehydrating the ion [92]. Furthermore, the cation permeability of the channel is relatively large (P_Na_/P_Cl_ = 0.1), and the channel is measurably, although weakly, permeant to NMDG^+^ (P_NMDG_/P_Cl_ = 0.06). We have modeled the pore as a dielectric tunnel with ɛ = 21 that exhibits some charge selectivity. These data reinforce the idea that the TMEM16A pore has features qualitatively similar to TMEM16F and that Cl^−^ ions traverse the evolutionary remnants of the phospholipid pathway.

Interestingly, TMEM16A reconstituted into liposomes composed of *E. coli* polar lipids and PtdCho exhibits very high selectivity to Cl^−^ relative to K^+^ [112]. Although these authors did not examine the relative permeability to various cations quantitatively, it appears that the reconstituted channel has different selectivity properties than the channel expressed in HEK cells. Whether this is related to the lipid composition or is some feature of the protein or its potential accessory subunits remains to be seen. Although there is the possibility that some of the cation permeability of TMEM16A/B observed in cells could be explained by contaminating currents carried by endogenous channels, this is not obviously supported by any data.

### TMEM16A has multiple conducting conformations

Another important feature of TMEM16A channels is that their permeability ratio changes with time after initiating whole-cell recording and with different [Ca^2+^], as if the channel has multiple open states [58, 126]. For example, P_I_/P_Cl_ = 11 immediately after raising cytosolic Ca^2+^, but decreases to 7.6 at later times. Furthermore, the relative ion permeabilities are dependent on Ca^2+^ concentration. For example, P_SCN_/P_Cl_ decreases from ~12 at 400 nM Ca^2+^, to <6 at 1.7 μM Ca^2+^. Similar results have been obtained with TMEM16B [8, 95]. Therefore, it appears that the TMEM16A channel has restricted and dilated conducting states. The presence of multiple conducting states with different biophysical properties could explain why different labs report quantitatively different anion permeability ratios for TMEM16A. If the channel exists in multiple conducting states, it suggests that the pore is rather flexible. Such flexibility might be facilitated by a proteolipidic pore.

One of the most intriguing aspects of CaCCs is that their biophysical properties are dependent on the Ca^2+^ concentrations used to activate them [59]. At low Ca^2+^ concentrations, the currents strongly rectify outwardly and exhibit voltage and time dependence, while they are essentially voltage-independent at higher Ca^2+^ concentrations. So striking is this plasticity that, when we first began studying these channels in the mid-1990s, we and others wondered whether CaCCs were comprised of two channels: one channel activated at low Ca^2+^ and another that became active at higher Ca^2+^ [14, 15, 39, 58]. In addition to the different biophysical properties of currents activated by different Ca^2+^ concentrations, the pharmacological properties (sensitivity to A9C) and regulation by PKC was reported to be Ca^2+^-dependent (see [59]). It is likely that this gating behavior is allosterically related to pore dilation. Interestingly, the crystal structure of nhTMEM16 reveals 2 Ca^2+^ ions in each monomer binding site [17]. We have previously shown that the Hill coefficient for current activation by Ca^2+^ increases from 1 at negative potentials to 2 or greater at positive potentials [92, 125]. This raises the possibility that the different conducting/gating states of the channel are related to the number of liganded Ca^2+^ ions.

Although other channels also have multiple conducting conformations, TMEM16A is unusual in the qualitative differences in current properties at different Ca^2+^ concentrations. For example, the conformational changes that occur during KcsA gating are very modest, largely because the way the protein is constructed and anchored in the membrane makes the selectivity filter relatively rigid. This assures that the backbone carbonyl oxygens are positioned at optimal distances to stabilize the permeant K^+^ ion. However, the selectivity filter of TMEM16A appears to be much more flexible.

### TMEM16A blockers are hydrophobic and a little weird

**Fig. 7:**
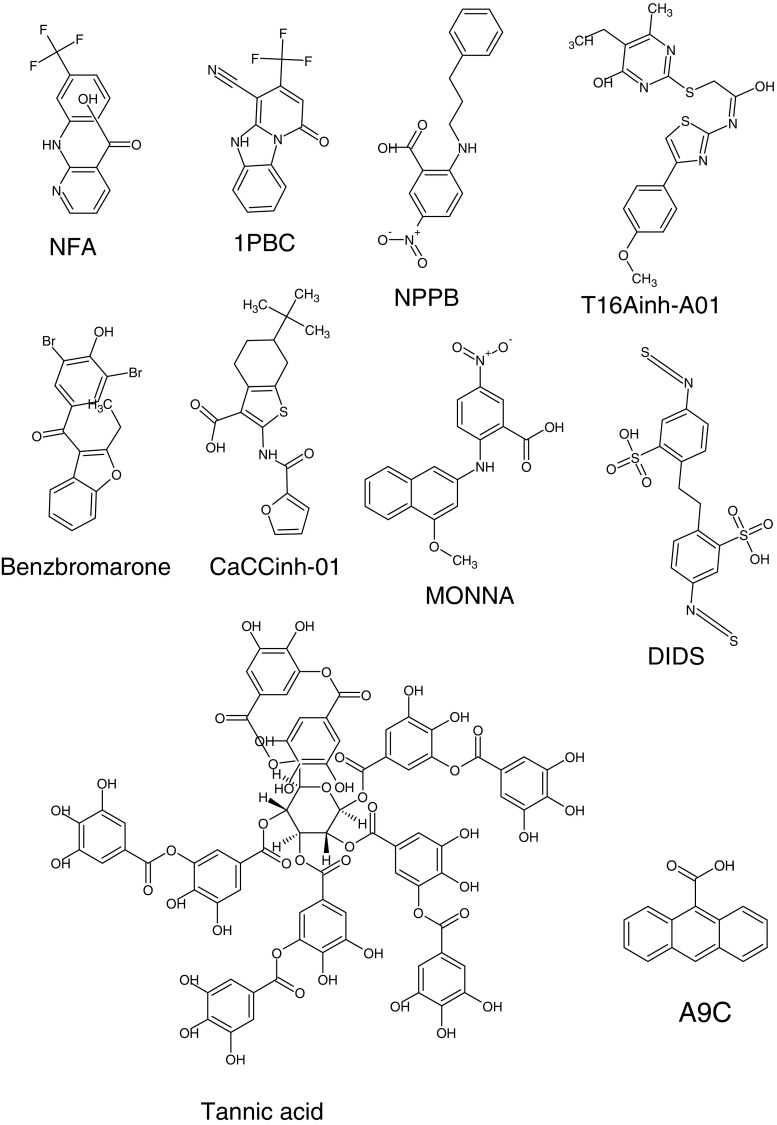
TMEM16A blockers are hydrophobic molecules. The structures include the classical TMEM16A blockers niflumic acid (NFA), anthacene-9-carboxylic acid (A9C), 4,4′-diisothiocyanostilbene-2,2′-disulfonic acid (DIDS), and 5-nitro-2-(3-phenylpropylamino)benzoic acid (NPPB) [38], and more recently identified inhibitors 1PBC [86], T16A_inh_-A01 [79], benzbromarone [49], CaCC_inh_-A01 [26], MONNA [83], and tannic acid [80]. Other blockers not shown are also hydrophobic structures with aromatic rings

Figure 7 shows the structures of some of the most common drugs used to block TMEM16A currents. In all cases, these compounds have at least two aromatic rings and are quite hydrophobic. Although this is a common feature of blockers of all Cl^−^ channels, not just TMEM16A channels, this suggests that these drugs act in a very hydrophobic domain of the protein, or alternatively, associated lipid. In general, these blockers have IC_50_’s in the tens of micromolar range. Blockers that have a potency in the low micromolar range seem to be only partly effective [66]. These large IC_50_’s suggest that the drugs do not have a high affinity binding site in the protein. The mechanisms of channel block have not been unambiguously elucidated for any of these compounds, and it remains unclear whether they block by acting in the permeation pathway or allosterically.

In cases where the pharmacology has been investigated in detail, these blockers have peculiar properties and do not behave like the classical cation channel blockers that we have come to love. For example, although A9C applied to the bath of whole cells behaves as a classic open pore blocker with voltage and time dependence, it paradoxically also blocks at apparently the same site when applied from the cytosolic side in inside-out patches [93]. The same is true of DPC. This suggests that although these drugs do not permeate the channel appreciably, they have access to the blocking site regardless of the side to which they are applied. The classic CaCC blocker NFA is even weirder. It blocks from either the outside or inside with little or no voltage dependence. This would seem to suggest that it blocks allosterically rather than by lodging in the pore. However, block by NFA is reduced by occupancy of the pore by permeating anions [82], which is characteristic of a blocker that competes with the permeant ion in the conduction pathway. Therefore, how does NFA access its binding site from both sides of the membrane and also compete with the permeant ion? Moreover, it has been reported that NFA and A9C have different effects on inward and outward currents. While NFA blocks outward currents, it slows the voltage-dependent inward deactivating inward tail currents [16].

Perhaps, the most puzzling blockers are polyphenols like tannic acid [80, 81]. These very hydrophobic molecules are much too large to enter the pore of the channel. Although they could sit on top of the permeation pathway, it remains uncertain how the hydrophobic tannins would dock on the hydrophilic surface of the channel. More likely, the tannins are binding to lipids. It is known that tannins have both stabilizing and destabilizing effects on model phospholipids and cellular membranes [121]. For example, tannins destabilize phospholipid bicelles by increasing the hydrophobic volume and reducing positive curvature to promote bicelle-to-hexagonal transition [32].

The explanations of these strange effects are not clear, but they challenge the idea that these compounds have a single specific binding site in the TMEM16A protein. The difficulty in interpretation is augmented by the fact that there appears to be serious quantitative disagreement in the literature about the potency of various blockers. The quantitative discrepancies can be illustrated with NFA. NFA has been reported to block native TMEM16A currents with Ki’s of 3–50 μM. Although these differences might be explained by splice variants or animal species, this is not the whole story. One possible explanation is that these drugs inhibit the channel, not by interacting *only* with protein, but by interacting with lipid. If these blockers act on lipids at sites of lipid-protein interaction, variations in drug efficacy and potency might be explained by differing membrane lipid compositions.

### TMEM16A single channels are not well characterized

Studies in native tissue (*Xenopus* oocytes and arterial smooth muscle) indicate that TMEM16A single channel conductance (γ) is ~3 pS with subconductance states [25, 38, 111]. The single channel conductance of recombinant TMEM16A is *γ* = 3.5 pS measured either by single-channel recording [19] or by noise analysis [1]. These reports conflict with Yang et al. who reported that *γ* = 8 pS [127]. However, none of these published data on TMEM16A single channels are compelling. There have been no studies showing that mutations in TMEM16A affect single channel conductance or gating; therefore, it remains an open question whether these single channels are actually encoded by TMEM16A or possibly are encoded by upregulated endogenous channels. We made a serious effort over several years to record TMEM16A single channels, and our impression is that the channels exhibit multiple low conductance states (0.5–3 pS) that are hard to resolve as discrete opening events and often look like noise. When we found discrete single channels, these were also present in native untransfected HEK cells. The published single channel traces confirm our experience: the traces are generally noisy (not surprising given the low single channel conductance) with multiple sub-conductance states [19]. Although amplitude histograms reveal discrete peaks, these histograms were apparently obtained from idealized traces or records filtered at a considerably lower frequency than the representative traces shown. Data from noise analysis are not convincing [1] because the variance vs. current amplitude plot does not reach a clear maximum, a requirement for extracting γ, and an observation we confirm. The paucity of data on TMEM16A single channel behavior is another limitation in understanding how these channels operate.

### The Role of Lipids in Ion Channel Pores

Although the role of lipids in ion channel structure and function is becoming more widely appreciated [5, 6, 37, 44, 61, 89, 110], the participation of lipids in the conduction pathway has not been previously suggested to our knowledge. However, the idea that lipids can participate in forming hydrophilic pores in lipid membranes is well-known. Pure lipid membranes can form hydrophilic pores, especially near lipid phase transition temperatures. This can be facilitated by membrane proteins that are unable by themselves to form channels [41, 78]. Pure lipidic pores can exhibit quantized single channel currents that are very similar to ion channel protein single channel currents, except that the lipid pore conductances are frequently much larger. Interestingly, these channels observed in membranes made of cationic lipids are often anion-selective. One way that lipid pores are thought to occur is by formation of non-lamellar structures in the membrane.

Many small pore-forming peptides (PFPs) form aqueous pores by deforming the membrane to elicit the fusion of the inner and outer leaflets creating a proteolipidic channel (reviewed in [35]). These structures are known to form both “matrix-type” toroidal pores formed by interspersed polypeptide chains and lipids and “arc-type” pores where one half of the pore is formed by peptide and the other half is complemented by a toroidal arrangement of lipid head groups [73, 90, 91, 103]. The formation of toroidal pores is a major function of many antimicrobial peptides in combating bacteria, but it is now recognized that the amphipathic regions of many cellular proteins can also insert into and deform membranes resulting in toroidal pore formation in a manner analogous to the mechanism of pore formation in pure lipid membranes elicited by electrical charge or detergents [94, 116]. Many toroidal pores exhibit anionic selectivity. However, changes in the lipid composition of the membrane can greatly alter the ion selectivity of these structures, in some cases from anion- to cation-selective [3, 18, 101, 102]. Interestingly, many descriptions of toroidal pores are accompanied by observations of PLS occurring concurrently with ion conductance [34, 73, 74, 101, 117].

We are not suggesting that TMEM16s form toroidal pores, but we believe there are many parallels that can be drawn from the function of these proteolipidic structures and the hydrophilic furrow in TMEM16 proteins. If lipid head groups compose part of the TMEM16 pore, this suggests that the abundance of different lipid species in the protein’s resident membrane may contribute to ion selectivity. This could explain the variability observed in TMEM16A ion selectivity from different laboratories, as lipid head groups of various diameters would likely stabilize alternative dilation states of the TMEM16A pore discussed earlier.

## Conclusion

The ionic selectivity of ion channels is a macroscopic manifestation of the competing interactions between dissolved ions, water, and larger molecular units. In general, our understanding of how anions interact in this context remains less well-developed than that of cations. It may be a truism to say that Cl^−^ ions have different physicochemical properties than cations, but the differences extend beyond simple electronegativity. The electron affinities of anions are less than those of comparable cations or neutrals [4, 47] and the Cl^−^ ion is considerably larger than its cation counterparts that are relevant in biology (Na^+^, K^+^, Mg^2+^, and Ca^2+^). Not only does this mean that the pore diameter of Cl^−^ channels is likely to be larger, the lower charge density of a Cl^−^ ion is likely to make electrostatic interactions less influential. To overly simplify this point, a single charge distributed over the surface of Na^+^ ion of radius 1 Å will have a surface charge density ~3 times greater than a Cl^−^ ion with a radius of 1.8 Å. This opens the door to selectivity filter designs that operate in ways that differ from those we have come to expect from some cation channels. The reader may decide that our proteolipid pore hypothesis is a very pore idea. However, we believe that this idea is tenable, based on the *in flagrante* liason of lipids with many TMEM16 proteins and what we know about the conductance pathways of TMEM16A and TMEM16F. Furthermore, this proposal may help explain some of the enigmatic features of CaCC currents that have plagued us since early days. Understanding the relation of TMEM16s to lipids and the architecture of their resident membranes is essential to the elucidation of the mechanistic function of CaCCs and should be a major focus of future TMEM16 investigation.

### Disclaimer

Some of the generalizations made here do not take into consideration differences between (1) species, (2) splice variants, (3) native vs. cloned channels. It was assumed that native CaCC currents, like those in *Xenopus* oocytes are encoded by homomeric TMEM16A. All numbering is adjusted to mouse TMEM16A(a,c) sequence, even if experiments were performed on other splice variants or species with different numbering.

## Abbreviations

PLS: Phospholipid scrambling
Ca^2+^-PLS: Ca^2+^-dependent phospholipid scrambling
PtdSer: Phosphatidylserine
PtdCho: Phosphatidylcholine
NMDG^+^: *N*-Methyl d-glucamine
NFA: Niflumic acid
A9C: Anthracene 9-carboxylic acid
